# Retraction Note: Global energy use and carbon emissions from irrigated agriculture

**DOI:** 10.1038/s41467-026-77951-w

**Published:** 2026-09-22

**Authors:** Jingxiu Qin, Weili Duan, Shan Zou, Yaning Chen, Wenjing Huang, Lorenzo Rosa

**Affiliations:** 1 State Key Laboratory of Desert and Oasis Ecology, Key Laboratory of Ecological Safety and Sustainable Development in Arid Lands, Xinjiang Institute of Ecology and Geography, Chinese Academy of Sciences, Urumqi, 830011 China; 2 University of Chinese Academy of Sciences, Beijing, 100049 China; 3 Akesu National Sation of Observation and Research for Oasis Agro-ecosystem, Akesu, Xinjiang 843017 China; 4 North China University of Water Resources and Electric Power, Zhengzhou, 450046 China; 5 Department of Global Ecology, Carnegie Institution for Science, Stanford, CA 94025 USA

Retraction to: *Nature Communications* https://doi.org/10.1038/s41467-024-47383-5, published online 10 April 2024

This article was retracted because it was confirmed that, contrary to the stated methodology in the article, the authors used the GCWM instead of the WATNEEDS model. Weili Duan has stated on behalf of the authors that they do not agree to this retraction.

